# Benchmarking large language models on a Chinese radiation oncology technology examination-preparation question set: accuracy, consensus, and efficiency for AI assisted education

**DOI:** 10.3389/fonc.2026.1946351

**Published:** 2026-09-23

**Authors:** Heling Zhu, Yongguang Liang, Xinyu Long, Yanchen Liu, Tingtian Pang, Wenbo Li, Bo Yang, Lin Yao

**Affiliations:** 1 Department of Radiation Oncology, Peking Union Medical College Hospital, Chinese Academy of Medical Sciences and Peking Union Medical College, Beijing, China; 2 Huayi Boao (Beijing) Quantum Technology Co., Ltd., Beijing, China

**Keywords:** Chinese national examination, examination preparation, large language models (LLM), medical education, radiation oncology technology

## Abstract

**Purpose:**

This study evaluated GPT-4o, GPT-5.4, and two DeepSeek platform configurations using 1, 053 examination-preparation questions from a publicly and commercially available 2025 exercise collection for the Chinese National Radiation Oncology Technology Qualification Examination (Intermediate Level). We assessed accuracy rates, identical-response coverage, accuracy consensus, and model-reported latency estimates.

**Methods:**

Four configurations—GPT-4o, GPT-5.4, DS-Fast, and DS-Expert—were tested using an identical zero-shot Chinese prompt. Accuracy rates were summarized with Wilson 95% confidence intervals. Pairwise differences in the primary Chinese-language analysis were assessed using exact McNemar’s tests with Holm adjustment. A descriptive language-controlled analysis evaluated English translations of the same questions using an equivalent English prompt.

**Results:**

Overall accuracy rates were 56.7% (95% CI, 53.7–59.7) for GPT-4o, 66.1% (63.2–68.9) for GPT-5.4, 98.4% (97.4–99.0) for DS-Fast, and 99.8% (99.3–99.9) for DS-Expert. All six overall pairwise comparisons remained significant after Holm adjustment. DS-Fast and DS-Expert produced identical responses for 1, 034 of 1, 053 questions (98.2%), all of which were correct. GPT-5.4 and DS-Expert showed 100% conditional accuracy among shared responses, but their identical-response coverage was only 65.9% (694/1, 053). Model-reported latency estimates were lowest for DS-Fast (0.68 ± 1.00 seconds) and highest for DS-Expert (3.69 ± 2.24 seconds). Under the English-translated condition, overall accuracy rates were 62.6%, 61.5%, 66.5%, and 76.9%, respectively. DS-Expert remained the most accurate configuration, although its advantage over the GPT models was reduced.

**Conclusion:**

DeepSeek configurations outperformed the GPT models on this Chinese-language examination-preparation question set, with DS-Expert achieving the highest accuracy. However, performance varied substantially by question language. These findings support further evaluation for answer verification and practice-question review, but they do not establish educational effectiveness.

## 1 Introduction

In recent years, large language models (LLMs) have demonstrated remarkable capabilities in natural language understanding, reasoning, and knowledge synthesis, attracting increasing attention regarding their potential applications in healthcare (1–3). Previous studies have evaluated LLM performance on standardized medical examinations, encompassing directions such as clinical decision support and medical education assistance (4–10). Multicenter studies have further identified the barriers and facilitators influencing LLM adoption in clinical settings, highlighting the need for systematic evaluation of AI integration in healthcare (11). However, radiation oncology and medical physics are highly specialized technical fields where clinical practice demands extreme precision. Therefore, the integration of LLMs into these domains necessitates rigorous benchmarking against authoritative standards such as professional qualification examinations.

The application of LLMs in radiation oncology education and assessment has garnered increasing research attention. GPT-4o and GPT-5.4 (OpenAI, CA, USA) are two generations of internationally developed LLMs built on Transformer architecture and reinforcement learning from human feedback (12, 13). Previous studies have demonstrated that GPT-4o performs excellently in medical knowledge evaluation: achieving 78.77% accuracy rate on the American College of Radiology Radiation Oncology In-Training Examination (14) and 77% accuracy rate on the RAPHEX radiation physics examination (15). These results indicate that current internationally developed LLMs such as GPT possess a certain degree of professional knowledge in radiation oncology and medical physics. Notably, however, existing evaluations have predominantly focused on English-language examination scenarios. Although modern frontier LLMs are multilingual, their performance may vary across languages, specialties, and examination contexts. The challenges of LLM performance in non-English medical contexts have been further documented, with qualitative studies revealing significant variations in model accuracy rates across different language settings (16). For non-English medical examination tasks, Takagi et al. evaluated the performance of GPT-3.5 and GPT-4 on the Japanese Medical Licensing Examination (JMLE), finding that GPT-4 achieved an overall accuracy rate of 79.9%, significantly outperforming GPT-3.5’s 50.8%, and met the passing criteria across all question types (17). Similarly, Oh et al. compared GPT-3.5 and GPT-4 on the Korean General Surgery Board Examination, with results showing that GPT-4 achieved an overall accuracy rate of 76.4%, representing a substantial improvement over GPT-3.5’s 46.8% (18). These two studies demonstrated the significant performance gains of GPT-4 over its predecessor in non-English medical examinations. More recently, Zhu et al. reported that GPT-4o further improved upon GPT-4 on the Chinese National Nursing Licensing Examination, achieving 77.1% accuracy rate compared to GPT-4’s 72.5% (P< 0.001), with both generations surpassing the minimum passing score (19). Collectively, these studies demonstrate that the new generation of GPT models exhibits enhanced linguistic adaptability and professional reasoning capabilities than their predecessors. Based on this evidence, the present study selected the latest GPT-5.4 and GPT-4o, which is widely adopted in current research, as representatives of the GPT model series. It conducts comparisons between these two generations of models, as well as direct comparisons with Chinese-developed models.

DeepSeek (Hangzhou DeepSeek AI, Zhejiang, China) is a Chinese-developed LLM family. Previous research related to Chinese medical examinations has shown that DeepSeek-R1 (20) delivers outstanding performance compared with the GPT series of models. In the Chinese Medical Licensing Examination, the study by Wang et al. provided direct evidence for this language dependency (21): DeepSeek-R1 achieved 96% accuracy rate, significantly surpassing GPT-4o’s 75.3%. Moreover, when the examination questions were translated into English, GPT-4o’s accuracy rate significantly improved from 75.3% to 83.5%, yet remained significantly lower than DeepSeek-R1’s native Chinese performance. The same issue is also reflected in research on Taiwan’s Family Medicine Board Exam conducted using GPT (22). This “translation gain” phenomenon directly confirms the substantial constraints imposed by language barriers, while also suggesting that Chinese-developed models may possess unique advantages in domestic medical examinations.

Although the aforementioned studies provide important references for the application of LLMs in Chinese-based general medical examinations, radiation oncology technology possesses distinctive characteristics: this field requires not only mastery of complex radiation physics principles and biological effects but also involves precise equipment operation, dosimetric calculations, and quality assurance procedures—knowledge that is highly dependent on Chinese domestic textbooks and clinical guidelines. Existing evaluations of LLMs on the Chinese Radiation Oncology Technology Qualification Examination (Intermediate Level) remain limited. Therefore, this study aims to evaluate the performance of GPT-4o, GPT-5.4, and two configurations of DeepSeek using the officially recommended 2025 examination-preparation question set for the Chinese Radiation Oncology Technology Qualification Examination. By comparing accuracy rates, identical-response coverage, accuracy consensus, and model-reported latency estimates, this study provides empirical evidence regarding the feasibility of LLMs for examination-answering support in radiation oncology technology education.

## 2 Materials and methods

### 2.1 Large language models

Four model configurations were evaluated in this study: GPT-4o, GPT-5.4, DS-Fast, and DS-Expert. GPT-4o and GPT-5.4 are OpenAI-developed general-purpose multilingual models. DS-Fast and DS-Expert are platform-level configurations provided by the DeepSeek platform rather than independently deployable model variants. According to the DeepSeek-V4 technical report (23), DS-Fast is powered by DeepSeek-V4-Flash, whereas DS-Expert is powered by DeepSeek-V4-Pro. DS-Fast is intended for conventional question-answering tasks and prioritizes rapid responses, whereas DS-Expert is designed for complex reasoning tasks, including mathematics, physics, programming, and logical analysis. In this study, the two DeepSeek configurations were evaluated separately to examine whether the two DeepSeek configurations differed in accuracy rates on radiation oncology technology examination-preparation questions.

The model version dates were December 15, 2025 for GPT-4o; March 5, 2026 for GPT-5.4; and April 24, 2026 for both DeepSeek-V4-Flash and DeepSeek-V4-Pro. The Chinese-language evaluation was conducted on May 25, 2026, and the English-translated evaluation was conducted on August 21, 2026. The same model versions were used for both language conditions.

### 2.2 Examination and input source

The Chinese Radiation Oncology Technology Qualification Examination is a component of the Chinese National Health Professional Qualification Examination (Intermediate Level), designed to systematically evaluate the professional knowledge and practical operational competencies of radiation therapy technologists and physicists. The examination comprises four subjects: Basic Knowledge, Relevant Professional Knowledge, Professional Knowledge, and Professional Practical Ability. Basic Knowledge covers General Introduction, Fundamentals of Radiotherapy Physics, and Biology; Relevant Professional Knowledge emphasizes the clinical characteristics and treatment principles of common tumors, including Head and Neck Tumors, Thoracic Tumors, Abdominal Tumors, and Cervical Cancer; Professional Knowledge focuses on Machines and Auxiliary Equipment, Process, Irradiation Techniques, Field Design, Intensity-Modulated Radiotherapy (IMRT) and Stereotactic Radiotherapy (SRT) Techniques, and Quality Assurance; Professional Practical Ability primarily examines the Responsibilities of Radiation Technicians, and Techniques of Simulation and Positioning.

The 1, 053 questions analyzed in this study were obtained from the Radiation Oncology Technology section of the 2025 edition of Oncology and Radiation Oncology Technology: Synchronized Exercise Collection published by People’s Medical Publishing House (ISBN 978-7-117-37350-0) (24). This book is an officially recommended and publicly available examination-preparation collection in the National Health Professional Technical Qualification Examination exercise series. The analyzed questions included 256 items on basic knowledge, 175 on relevant professional knowledge, 408 on professional knowledge, and 214 on professional practical ability. The reference answers printed in the same published volume were used as the scoring key. The questions were used solely as inputs for model evaluation, and no question bank or item-level question content was reproduced in the manuscript or publicly redistributed. All evaluated items were text-based, single-best-answer multiple-choice questions. Each item consisted of one question stem followed by five answer options labeled A through E, with exactly one correct option according to the publisher-provided reference answers.

### 2.3 Questioning process

GPT-4o, GPT-5.4, and two DeepSeek configurations (DS-Fast vs. DS-Expert) were queried using a standardized zero-shot prompt in Chinese (English translation provided below), which instructed the model to assume the role of a certified technician and to restrict output exclusively to answer identifiers and a model-reported latency estimate:

“You are a professional radiation oncology technician currently taking the Radiation Oncology Technology Qualification Examination (Intermediate Level). Please answer the following questions accurately based on your professional knowledge.

Rules:
1. Provide only the answer letter, all questions are single-choice questions.
2. Do not provide explanations, supplementary information, or line breaks.
3. The thinking time for this response must be appended at the end in the following format: [Time: X.XXX seconds].
4. Adhere strictly to the standard answer key; do not modify or reinterpret the information provided in the question.

Please answer now:

(Paste question and options here)”

All four model configurations were accessed through the official OpenAI and DeepSeek APIs. The identical standardized prompt was used for all questions and all four configurations. No system or developer message was included in any request, no additional safety instructions or non-default settings were configured by the authors, and all API default parameters were retained.

### 2.4 Evaluation metrics

Three answer-based metrics—accuracy rate, identical-response coverage, and accuracy consensus—and one exploratory descriptive metric, model-reported latency estimate, were used to evaluate model performance.

#### 2.4.1 Accuracy rate

was calculated as the proportion of questions answered correctly by a model relative to the total number of test items, expressed as:


$$\text{Accuracy rate}=\frac{\text{Number of correct responses}}{\text{Total number of questions}}$$

A response was scored correct if it exactly matched the reference answer; all mismatches, omissions, extra selections, and non-responsive outputs were scored as incorrect.

#### 2.4.2 Identical-response coverage

was used as a descriptive measure of raw overall agreement between two models, which was calculated as:


$$\text{Identical}-\text{response coverage}=\frac{\text{Number of questions on which both models gave identical responses}}{\text{Total number of questions}}$$

This metric indicates the breadth of agreement between two models across the full question set.

#### 2.4.3 Accuracy consensus

was used as a conditional measure of answer correctness when two models returned the same response. Following Wang et al. (21), accuracy consensus was calculated as:


$$\text{Accuracy consensus}=\frac{\text{Number of questions both models answered correctly}}{\text{Number of questions with identical responses from both models}}$$

This metric reflects the reliability of shared correct judgments and helps readers evaluate whether using two large language models simultaneously can yield more reliable results.

#### 2.4.4 Model-reported latency estimate

was defined as the numeric time value generated by each model in response to the prompt instruction, expressed in seconds and recorded to three decimal places. This metric was analyzed as an exploratory descriptive metric.

### 2.5 Language-controlled analysis

To evaluate the sensitivity of model performance to question language, all 1, 053 Chinese questions, including the stems and response options, were translated into English item by item using Volctrans (ByteDance, Beijing, China) and reviewed by a senior Physicist before model evaluation. No additional paraphrasing, option reordering, content correction, or answer-key modification was performed. Item order, response-option structure, and the publisher-provided reference answers were retained. The English-translated items were then presented to the same four model configurations using the same prompt structure, model settings, and correctness-scoring procedures as in the primary Chinese-language analysis. Accuracy rate was calculated overall and by subject. This analysis was descriptive and was intended to assess sensitivity to question language rather than to infer training-corpus composition or causal mechanisms.

### 2.6 Statistical analysis

Pairwise differences in accuracy rates between model configurations were assessed using two-sided exact McNemar’s tests based on the numbers of discordant responses. For each comparison, the numbers of items answered correctly by only one model were recorded. For each model configuration and question category, 95% confidence intervals for accuracy rates were calculated using the Wilson score method. Confidence intervals were reported for overall, subject-level, and subsection-level accuracy rates estimates in the primary Chinese-language analysis.

To account for multiple testing while preserving the hierarchical structure of the analysis, comparisons were divided into three prespecified families: (1) six overall pairwise comparisons among the four model configurations; (2) 24 subject-level comparisons, comprising six model pairs across four examination subjects; and (3) 90 subsection-level comparisons, comprising six model pairs across 15 subsections. Within each family, P-values were adjusted using the Holm procedure, which controls the family-wise error rate. Holm-adjusted P-values are reported, and statistical significance was interpreted using a two-sided adjusted P-value<0.05. Statistical analyses were performed using Python 3.10.9.

## 3 Results

### 3.1 Accuracy rates

The overall accuracy rates of GPT-4o, GPT-5.4, DS-Fast, and DS-Expert across 1, 053 questions were 56.7% (95% CI, 53.7–59.7), 66.1% (95% CI, 63.2–68.9), 98.4% (95% CI, 97.4–99.0), and 99.8% (95% CI, 99.3–99.9), respectively. GPT-5.4 demonstrated a meaningful improvement of 9.4 percentage points over GPT-4o; however, both GPT models remained substantially below the DeepSeek configurations. As previously described, all questions were divided into four subjects, each further subdivided into 3–5 different sections. The overall accuracy rates, subject-specific and section-specific accuracy rates, together with their 95% confidence intervals, are presented in **Table 1**. Holm-adjusted P-values for the overall and subject-level pairwise comparisons are presented in **Table 2**. All six overall pairwise comparisons remained statistically significant. GPT-5.4 showed significantly higher overall accuracy rate than GPT-4o (adjusted P = 3.900 × 10^-7^). Both DeepSeek configurations significantly outperformed both GPT models, and DS-Expert showed a small but statistically significant advantage over DS-Fast (adjusted P = 7.286 × 10^-4^). At the subject level, 18 of 24 pairwise comparisons remained significant after adjustment. All 16 comparisons between either GPT model and either DeepSeek configuration remained significant. GPT-5.4 outperformed GPT-4o in Basic Knowledge and Professional Practical Ability, but not in Relevant Professional Knowledge or Professional Knowledge. DS-Fast and DS-Expert did not differ significantly within any individual subject after adjustment. Holm-adjusted P-values for all subsection-level comparisons are provided in **Supplementary File S1**. **Figure 1** illustrates the overall accuracy rates of the four model configurations, accuracy rates across the four subject categories, and pairwise significance.

**Table 1:** Number of questions in each subject and subsection, as well as the accuracy rates and 95% confidence intervals of the four model configurations across different categories.

Question types	Total questions	GPT-4o accuracy rate, % (95% CI)	GPT-5.4 accuracy rate, % (95% CI)	DS-fast accuracy rate, % (95% CI)	DS-expert accuracy rate, % (95% CI)
**Overall**	**1053**	**56.7 (53.7–59.7)**	**66.1 (63.2–68.9)**	**98.4 (97.4–99.0)**	**99.8 (99.3–99.9)**
**Basic Knowledge**	**256**	**50.4 (44.3–56.5)**	**66.8 (60.8–72.3)**	**98.4 (96.1–99.4)**	**99.6 (97.8–99.9)**
General Introduction	27	85.2 (67.5–94.1)	85.2 (67.5–94.1)	100.0 (87.5–100.0)	96.3 (81.7–99.3)
Fundamentals of Radiotherapy Physics	137	48.9 (40.7–57.2)	53.3 (45.0–61.4)	99.3 (96.0–99.9)	100.0 (97.3–100.0)
Fundamentals of Radiotherapy Biology	92	42.4 (32.8–52.6)	81.5 (72.4–88.1)	96.7 (90.8–98.9)	100.0 (96.0–100.0)
**Relevant Professional Knowledge**	**175**	**81.1 (74.7–86.2)**	**78.3 (71.6–83.8)**	**97.1 (93.5–98.8)**	**100.0 (97.9–100.0)**
Head and Neck Tumors	60	75.0 (62.8–84.2)	75.0 (62.8–84.2)	98.3 (91.1–99.7)	100.0 (94.0–100.0)
Thoracic Tumors	56	82.1 (70.2–90.0)	82.1 (70.2–90.0)	98.2 (90.6–99.7)	100.0 (93.6–100.0)
Abdominal Tumors	51	86.3 (74.3–93.2)	76.5 (63.2–86.0)	94.1 (84.1–98.0)	100.0 (93.0–100.0)
Cervical Cancer	8	87.5 (52.9–97.8)	87.5 (52.9–97.8)	100.0 (67.6–100.0)	100.0 (67.6–100.0)
**Professional Knowledge**	**408**	**59.6 (54.7–64.2)**	**62.3 (57.5–66.8)**	**98.5 (96.8–99.3)**	**99.8 (98.6–100.0)**
Machines and Auxiliary Equipment	118	68.6 (59.8–76.3)	68.6 (59.8–76.3)	100.0 (96.8–100.0)	99.2 (95.4–99.9)
Radiotherapy Process	52	59.6 (46.1–71.8)	67.3 (53.8–78.5)	100.0 (93.1–100.0)	100.0 (93.1–100.0)
Irradiation Techniques and Field Design	48	58.3 (44.3–71.2)	66.7 (52.5–78.3)	93.8 (83.2–97.9)	100.0 (92.6–100.0)
IMRT and SRT	100	74.0 (64.6–81.6)	75.0 (65.7–82.5)	99.0 (94.6–99.8)	100.0 (96.3–100.0)
Quality Assurance	90	32.2 (23.5–42.4)	34.4 (25.4–44.7)	97.8 (92.3–99.4)	100.0 (95.9–100.0)
**Professional Practical Ability**	**214**	**38.8 (32.5–45.5)**	**62.6 (56.0–68.8)**	**99.1 (96.7–99.7)**	**100.0 (98.2–100.0)**
Responsibilities of Radiation Technician	37	86.5 (72.0–94.1)	83.8 (68.9–92.3)	100.0 (90.6–100.0)	100.0 (90.6–100.0)
Simulation Localization Techniques	76	26.3 (17.7–37.2)	59.2 (48.0–69.6)	100.0 (95.2–100.0)	100.0 (95.2–100.0)
Positioning Techniques	101	30.7 (22.5–40.3)	57.4 (47.7–66.6)	98.0 (93.1–99.5)	100.0 (96.3–100.0)

Bold values indicates overall performance and results for the four main subjects.

**Table 2:** Holm-adjusted McNemar’s P-values for pairwise comparisons of overall and subject-level accuracy rates.

Analysis level	GPT-4o vs GPT-5.4	GPT-4o vs DS-Fast	GPT-4o vs DS-Expert	GPT-5.4 vs DS-Fast	GPT-5.4 vs DS-Expert	DS-Fast vs DS-Expert
Overall	3.900 × 10^-7^	7.120 × 10^-118^	1.695 × 10^-132^	1.554 × 10^-87^	4.402 × 10^-103^	7.286 × 10^-4^
Basic Knowledge	3.879 × 10^-4^	1.007 × 10^-34^	1.365 × 10^-35^	1.845 × 10^-20^	3.373 × 10^-23^	1.000
Relevant Professional Knowledge	1.000	1.553 × 10^-5^	2.561 × 10^-9^	1.026 × 10^-6^	8.731 × 10^-11^	0.375
Professional Knowledge	1.000	6.561 × 10^-41^	8.570 × 10^-47^	1.121 × 10^-38^	1.571 × 10^-4^³	0.625
Professional Practical Ability	1.317 × 10^-6^	5.584 × 10^-38^	1.469 × 10^-38^	1.876 × 10^-21^	2.647 × 10^-23^	1.000

Subsection-level Holm-adjusted P-values are provided in **Supplementary File S1**.

**Figure 1:**
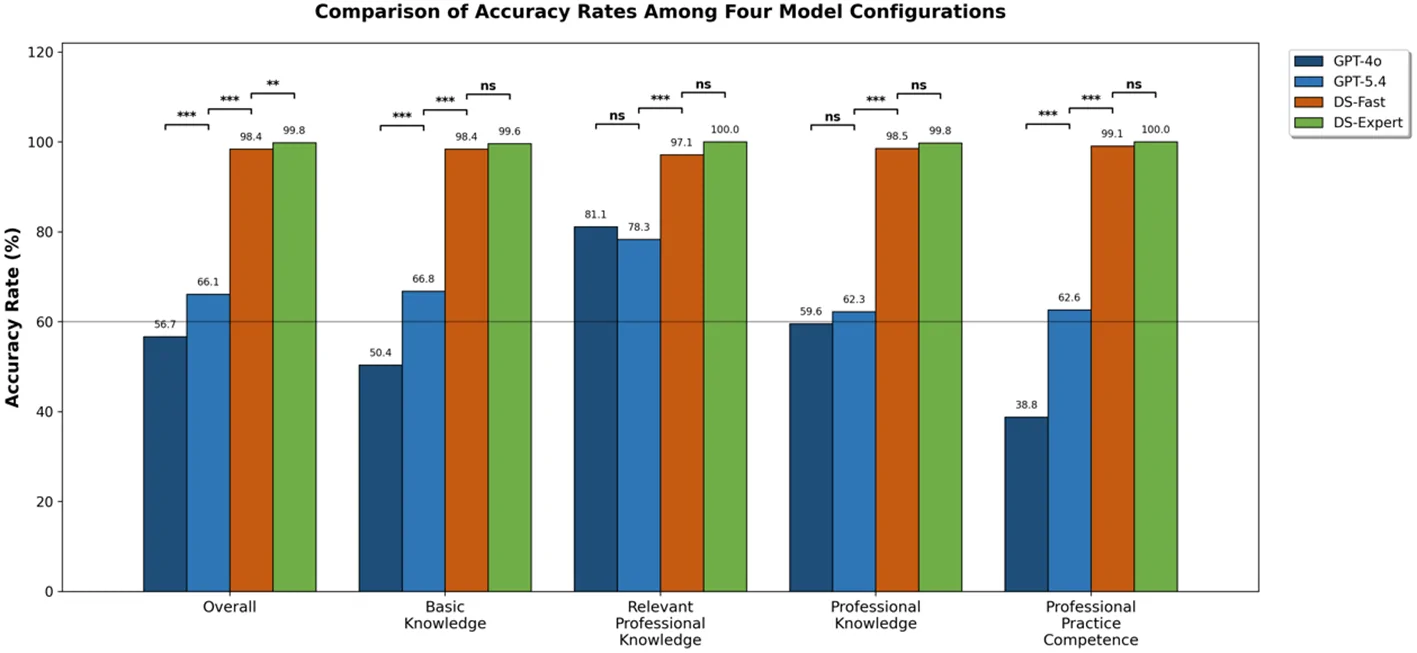
Accuracy rates of the four model configurations overall and across the four examination subjects. Pairwise differences were assessed using two-sided exact McNemar’s tests, with Holm adjustment applied within the corresponding prespecified testing family. Holm-adjusted P-values for the overall and subject-level comparisons are shown in **Table 2**; subsection-level results are provided in **Supplementary File S1**.

To characterize areas of greatest potential risk, incorrect-response rates were examined across examination subjects and subsections. Among GPT-4o responses, the highest error rates were observed in Simulation Localization Techniques (73.7%, 56/76), Positioning Techniques (69.3%, 70/101), and Quality Assurance (67.8%, 61/90). GPT-5.4 showed a similar pattern, with the highest error rates in Quality Assurance (65.6%, 59/90), Positioning Techniques (42.6%, 43/101), and Simulation Localization Techniques (40.8%, 31/76). In contrast, DS-Fast had error rates of no more than 6.2% across all subsections, with the highest rates observed in Irradiation Techniques and Field Design (6.2%, 3/48), Abdominal Tumors (5.9%, 3/51), and Fundamentals of Radiotherapy Biology (3.3%, 3/92). DS-Expert made only two errors among the 1, 053 questions: one in General Introduction (3.7%, 1/27) and one in Machines and Auxiliary Equipment (0.8%, 1/118). These findings indicate that Quality Assurance, Simulation Localization Techniques, and Positioning Techniques represent the highest-risk content areas for the GPT models in this question set.

### 3.2 Identical-response coverage and accuracy consensus

Because GPT-4o exhibited the lowest overall accuracy rate, **Table 3** focuses on agreement patterns involving GPT-5.4, DS-Fast, and DS-Expert. DS-Fast and DS-Expert showed high identical-response coverage, producing the same answer for 1, 034 of 1, 053 questions (98.2%), and all shared responses were correct, yielding an accuracy consensus of 100%. GPT-5.4 and DS-Expert produced identical responses for 694 questions (65.9%), all of which were correct, yielding an accuracy consensus of 100%. GPT-5.4 and DS-Fast produced identical responses for 689 questions (65.4%), of which 684 were correct, yielding an accuracy consensus of 99.3%. Across the four subjects, identical-response coverage for DS-Fast versus DS-Expert ranged from 97.1% to 99.1%, whereas coverage for the two cross-model pairs ranged from 61.5% to 78.3%. These results show that identical-response coverage and accuracy consensus capture different aspects of model agreement: the former reflects how often two models produced the same answer, whereas the latter reflects the correctness of that shared subset.

**Table 3:** Identical-response coverage and accuracy consensus for GPT-5.4, DS-Fast, and DS-Expert.

Question types		GPT-5.4 vs. DS-Fast				GPT-5.4 vs. DS-Expert				DS-Fast vs. DS-Expert		
	Both correct	Identical incorrect	Identical responses (%)	Accuracy consensus (%)	Both correct	Identical incorrect	Identical responses (%)	Accuracy consensus (%)	Both correct	Identical incorrect	Identical responses (%)	Accuracy consensus (%)
Overall	684	5	65.4	99.3	694	0	65.9	100.0	1034	0	98.2	100.0
Basic Knowledge	168	1	66.0	99.4	170	0	66.4	100.0	251	0	98.0	100.0
Relevant Professional Knowledge	133	1	76.6	99.3	137	0	78.3	100.0	170	0	97.1	100.0
Professional Knowledge	250	1	61.5	99.6	253	0	62.0	100.0	401	0	98.3	100.0
Professional Practical Ability	133	2	63.1	99.3	134	0	62.6	100.0	212	0	99.1	100.0

### 3.3 Model-reported latency estimates

**Table 4** and **Figure 2** summarize the model-reported latency estimates for the four model configurations. DS-Expert reported the highest average estimate (3.69 ± 2.24 seconds), followed by GPT-5.4 (1.83 ± 1.46 seconds), GPT-4o (1.49 ± 0.29 seconds), and DS-Fast (0.68 ± 1.00 seconds). Notably, all four models reported the latency estimates within 4 seconds on average, indicating that even the most computationally intensive configuration maintains a response speed adequate for real-time educational assistance. Because these values were generated by the models rather than recorded from platform timestamps, they should be interpreted only as exploratory descriptive estimates.

**Table 4:** Average model-reported latency estimates (seconds) of the four model configurations for answering questions across different subjects.

Question types	GPT-4o (s)	GPT-5.4 (s)	DS-Fast (s)	DS-Expert (s)
Overall	1.49 ± 0.29	1.83 ± 1.46	0.68 ± 1.00	3.69 ± 2.24
Basic Knowledge	1.49 ± 0.30	1.74 ± 1.06	0.58 ± 0.18	3.58 ± 2.20
Relevant Professional Knowledge	1.53 ± 0.30	1.90 ± 2.46	0.65 ± 0.40	3.60 ± 2.11
Professional Knowledge	1.50 ± 0.29	1.85 ± 1.27	0.66 ± 0.45	3.67 ± 2.12
Professional Practical Ability	1.47 ± 0.28	1.82 ± 1.08	0.90 ± 2.08	3.92 ± 2.58

**Figure 2:**
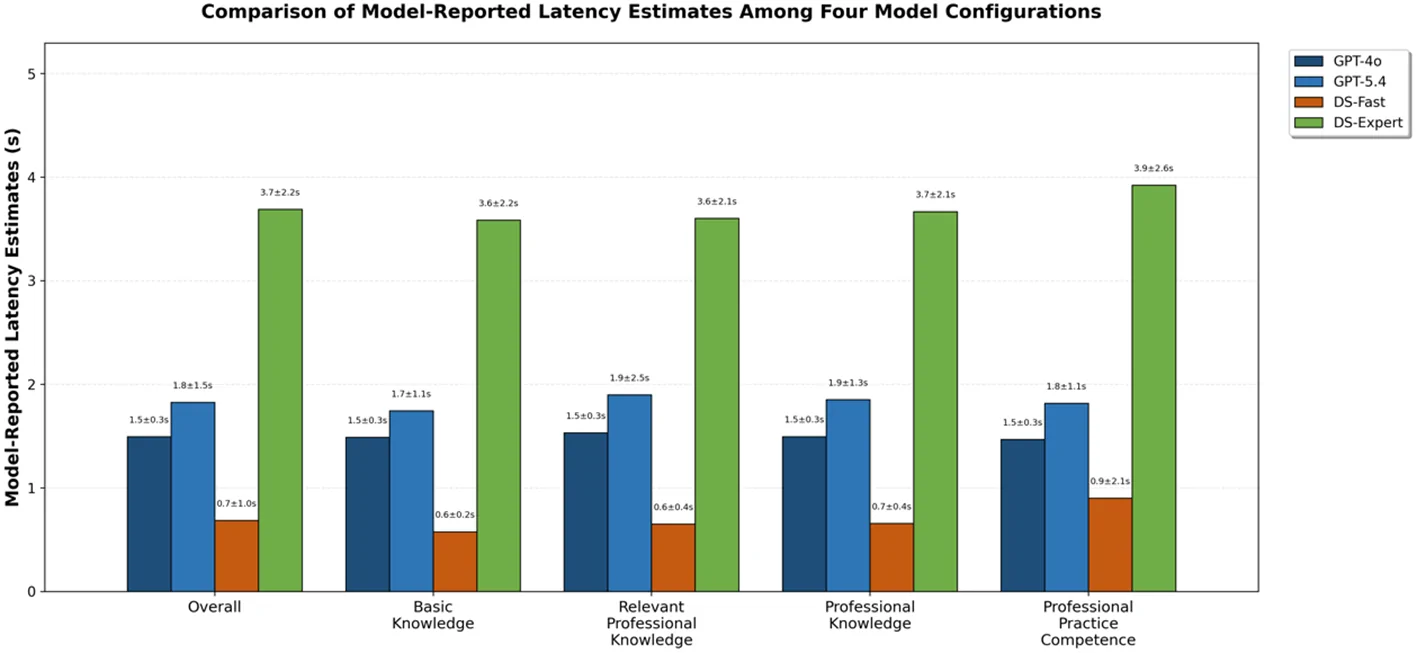
Average model-reported latency estimates of the four model configurations, overall and across the four examination subjects.

### 3.4 Language-controlled analysis

Under the English-translated condition, overall accuracies were 62.6% for GPT-4o, 61.5% for GPT-5.4, 66.5% for DS-Fast, and 76.9% for DS-Expert. Compared with the primary Chinese-language results, GPT-4o improved by 5.9 percentage points, whereas GPT-5.4, DS-Fast, and DS-Expert decreased by 4.6, 31.9, and 22.9 percentage points, respectively. DS-Expert remained the most accurate configuration under both language conditions and across all four subjects. However, its performance advantage was substantially smaller for the English-translated questions: the gap between DS-Expert and GPT-4o decreased from 43.1 percentage points in Chinese to 14.3 percentage points in English, and the gap between DS-Expert and GPT-5.4 decreased from 33.7 to 15.4 percentage points. Subject-level results are summarized in **Table 5**.

**Table 5:** Accuracy rates of the four model configurations on English-translated versions of the 1, 053 questions, overall and by subject.

Question types	Total questions	GPT-4o accuracy rate (%)	GPT-5.4 accuracy rate (%)	DS-Fast accuracy rate (%)	DS-Expert accuracy rate (%)
Overall	1053	62.6%	61.5%	66.5%	76.9%
Basic Knowledge	256	64.5%	62.1%	66.0%	69.5%
Relevant Professional Knowledge	175	74.9%	70.9%	79.4%	84.0%
Professional Knowledge	408	58.1%	58.3%	65.9%	78.4%
Professional Practical Ability	214	58.9%	59.4%	57.5%	77.1%

## 4 Discussion

This study adds to the limited literature by systematically comparing two DeepSeek configurations and two consecutive generations of internationally developed LLMs, GPT-4o and GPT-5.4, on an examination-preparation question set for the Chinese National Radiation Oncology Technology Qualification Examination (Intermediate Level). The principal findings reveal a striking performance advantage for DeepSeek over both GPT generations across all four examination subjects in the primary Chinese-language analysis, with DS-Expert achieving an overall accuracy rate of 99.8% compared to GPT-4o’s 56.7% and GPT-5.4’s 66.1%. These results extend prior evidence that language-related differences in LLM performance are context- and domain-dependent but also extend this understanding to the domain of radiation oncology technology.

The accuracy rate differential between DeepSeek and the GPT models observed in this study is larger than that reported for general medical examinations, although previous findings have been mixed. Wang et al. reported that DeepSeek-R1 achieved 96.0% accuracy rate versus 75.3% for GPT-4o on the Chinese Medical Licensing Examination, representing a gap of approximately 21 percentage points (21). In contrast, Tang et al. found a smaller difference between DeepSeek-R1 and GPT-4o on bilingual Chinese medical licensing examination questions (91.0% vs. 82.8%) and detected no significant main effect of language (25). Compared with both studies, the performance gap in the present study was substantially larger: 43.1 percentage points between DS-Expert and GPT-4o and 33.7 percentage points between DS-Expert and GPT-5.4. This pronounced discrepancy may reflect the highly specialized and localization-dependent nature of radiation oncology technology, which relies heavily on Chinese clinical guidelines, national quality-assurance standards, and locally adopted equipment protocols. Differences in question sources, model versions, and study endpoints may also contribute. Taken together, these findings suggest that the advantage of DeepSeek is domain- and task-dependent rather than uniform across Chinese medical assessments.

The language-controlled analysis provided direct evidence that benchmark performance was language-dependent and that the magnitude and direction of the language effect varied across model configurations. GPT-4o improved by 5.9 percentage points under the English-translated condition, whereas GPT-5.4, DS-Fast, and DS-Expert decreased by 4.6, 31.9, and 22.9 percentage points, respectively. Nevertheless, DS-Expert remained the most accurate configuration under both language conditions, indicating that its advantage was not entirely language-specific. These findings suggest that the Chinese-language results should not be attributed solely to stable specialty knowledge or used to infer training-corpus composition. Instead, performance may reflect interactions among language exposure, domain-specific knowledge representation, model architecture, and platform-level inference configuration.

The most pronounced performance discrepancy was observed in the Professional Practical Ability subject, where GPT-4o achieved only 38.8% accuracy rate, while GPT-5.4 showed marked improvement to 62.6%, compared to DS-Fast’s 99.1% and DS-Expert’s 100.0%. This subject specifically evaluates clinical operational competencies, including simulation localization techniques and patient positioning protocols, which are heavily dependent on institution-specific and nationally standardized procedural knowledge. The near-perfect Chinese-language performance of the DeepSeek configurations in this domain may reflect differences in language exposure, domain-specific knowledge representation, model architecture, or platform-level inference configuration. However, the substantial decrease observed under the English-translated condition indicates that this advantage was strongly affected by question language. The present study cannot determine the contribution of training-data composition because those details are not publicly documented. Conversely, two GPT models demonstrated its strongest relative performance in the Relevant Professional Knowledge subject, which covers the clinical characteristics and treatment principles of common tumors. This finding is consistent with previous studies showing that GPT-4o performs well on clinically oriented oncology content in English-language examinations (14). The relatively better performance in this subject may reflect the greater availability of English-language literature on tumor clinical characteristics and treatment protocols in GPT-4o’s training data, as cancer treatment guidelines are often published internationally.

The risk-of-error analysis has direct educational relevance. Errors by GPT-4o and GPT-5.4 were concentrated in procedure- and safety-relevant domains, including quality assurance, simulation localization, and patient positioning. These areas require precise operational knowledge, and incorrect responses may carry greater educational or clinical significance than errors in general factual recall. Therefore, GPT models should not be used for unsupervised answer verification in these domains without expert review. Although the subject- and subsection-level analyses identify domains in which model performance differed, they do not establish the mechanisms underlying individual errors. A formal error analysis would require prospectively collected model explanations and expert adjudication using a predefined taxonomy.

The combined analysis of identical-response coverage and accuracy consensus clarified two distinct aspects of agreement between model pairs. DS-Fast and DS-Expert showed both high overall agreement and high conditional accuracy rates: they produced identical responses for 1, 034 of 1, 053 questions (98.2%), and all shared responses were correct. This pattern suggests strong within-platform concordance between the two DeepSeek configurations. In contrast, GPT-5.4 and DS-Expert produced identical responses for only 694 questions (65.9%), and GPT-5.4 and DS-Fast for 689 questions (65.4%). The shared responses were almost always correct, with accuracy consensus values of 100.0% and 99.3%, respectively. These high values indicate that the relatively small subsets of questions on which the models agreed were answered correctly. Accuracy consensus may be more informative when no model achieves near-ceiling accuracy. In the present study, the near-perfect accuracy rate of DS-Expert limited the incremental discriminatory value of this conditional metric. Nevertheless, reporting identical-response coverage alongside accuracy consensus allows agreement-based answer verification to be interpreted more transparently: coverage reflects the breadth of agreement, whereas accuracy consensus reflects the reliability of the agreed responses.

The model-reported latency estimates showed descriptive differences across the four model configurations. DS-Fast had the lowest average estimate (0.68 seconds) while maintaining 98.4% accuracy rate, whereas DS-Expert had the highest accuracy rate (99.8%) and the highest average estimate (3.69 seconds). GPT-5.4 and GPT-4o had intermediate estimates of 1.83 ± 1.46 and 1.49 ± 0.29 seconds, respectively. However, because these values were generated by the models, they cannot establish network-dependent response speed, or computational cost. They should therefore be interpreted only as exploratory descriptive outputs recorded under the original experimental conditions.

In recent years, artificial intelligence has seen rapidly expanding applications throughout the entire process of medical education, including enrollment and admission, curriculum design, academic assessment, and clinical reasoning. As a transformative force in health professions education, it is gaining increasingly widespread recognition (26). The findings of this study carry several important implications for the integration of LLMs into radiation oncology technology education. First, the strong examination performance of DeepSeek suggests that it is a promising candidate for answer verification and practice-question review in Chinese radiation therapy training programs. This recommendation is limited to examination-answering support and should not be interpreted as evidence of improved teaching effectiveness or learner outcomes. Although these systems may potentially assist with explanation generation or feedback, such applications require separate validation of explanation fidelity, reasoning quality, and educational impact. Second, the significant performance disparity across examination subjects highlights the need for domain-specific evaluation when selecting LLMs for educational purposes. A model that performs well on general medical knowledge may still exhibit substantial weaknesses in specialized technical domains. Radiation oncology technology programs should prioritize LLMs that have demonstrated competency in their specific curricular content rather than relying on general medical examination benchmarks. Third, the high identical-response coverage and 100% accuracy consensus between DS-Fast and DS-Expert suggests that a single, well-configured DeepSeek model may be sufficient for most examination-answering and answer-verification applications in this field. However, for high-stakes scenarios such as certification examination preparation, the small accuracy rate differential between DS-Fast and DS-Expert (1.4 percentage points) may justify further evaluation of DS-Expert when maximal answer accuracy is desired for examination-answering support.

This study has several limitations that should be acknowledged. First, the evaluation was conducted using a standardized zero-shot prompting strategy without domain-specific fine-tuning. Although the identical prompt and default API settings were used for all model configurations, model performance may be sensitive to the exact wording of the prompt and may not generalize to alternative zero-shot prompts, few-shot prompting strategies, domain-specific prompt engineering, or retrieval-augmented settings. Second, this study evaluated only final-answer accuracy rates and did not assess reasoning quality, explanation fidelity, or the impact of model use on learner knowledge, skills, or educational outcomes. Therefore, the findings demonstrate examination-answering performance rather than validated pedagogical effectiveness. Although high answer accuracy rate may be a prerequisite for useful educational support, it is not sufficient evidence of teaching utility. Future learner-centered studies are needed to evaluate reasoning quality, explanation fidelity, user trust, and predefined educational outcomes before these models can be recommended as validated teaching tools. Third, although a descriptive risk-of-error analysis was added to identify content areas in which errors were concentrated, this analysis was based on final-answer correctness and could not determine the underlying mechanisms of individual errors. Because the standardized prompt restricted outputs to answer letters and model-reported latency estimates, model reasoning traces were not captured, preventing reliable classification of errors as knowledge gaps, comprehension failures, reasoning errors, or outdated guideline use. Future studies should prospectively collect model explanations and apply a predefined, expert-adjudicated error taxonomy to characterize failure mechanisms.

## 5 Conclusion

This study systematically evaluated GPT-4o, GPT-5.4, and two DeepSeek platform configurations (DS-Fast and DS-Expert) using a 2025 examination-preparation question set for the Chinese National Radiation Oncology Technology Qualification Examination (Intermediate Level). DS-Expert and DS-Fast achieved overall accuracy rates of 99.8% and 98.4%, respectively, substantially outperforming GPT-5.4 (66.1%) and GPT-4o (56.7%). DS-Fast and DS-Expert produced identical responses for 1, 034 of 1, 053 questions (98.2%), and all shared responses were correct. DS-Fast provided the lowest model-reported latency estimate (0.68 ± 1.00 s), whereas DS-Expert achieved the highest accuracy rate (99.8%) with an estimate of 3.69 ± 2.24 s. Under the English-translated condition, DS-Expert remained the most accurate configuration, although its advantage over the GPT models was substantially reduced. These findings support further evaluation of DeepSeek configurations for answer verification and practice-question review in radiation oncology technology education; their pedagogical effectiveness, reasoning quality, and impact on learner outcomes require prospective validation.

## Data Availability

The raw data supporting the conclusions of this article will be made available by the authors, without undue reservation.
