# Supplementary material for: Benchmarking large language models on a Chinese radiation oncology technology examination-preparation question set: accuracy, consensus, and efficiency for AI assisted education
Source: Front Oncol. 2026 Sep 23;16:1946351. doi: 10.3389/fonc.2026.1946351 (PMC13642277; doi:10.3389/fonc.2026.1946351)
Supplement: Supplementary file 1 [file Table1.docx]

**Supplementary File S1.** Holm-adjusted McNemar P-values and discordant-pair counts for all pairwise comparisons at the overall, subject, and subsection levels.

| **Chapter** | **Section** | **n** | **Model A** | **Model B** | **Exact McNemar P** | **Holm-adjusted P** |
| --- | --- | --- | --- | --- | --- | --- |
| Basic Knowledge | General Introduction | 27 | GPT-4o | GPT-5.4 | 1.000E+00 | 1.000E+00 |
| Basic Knowledge | General Introduction | 27 | GPT-4o | DS-Fast | 1.250E-01 | 1.000E+00 |
| Basic Knowledge | General Introduction | 27 | GPT-4o | DS-Expert | 3.750E-01 | 1.000E+00 |
| Basic Knowledge | General Introduction | 27 | GPT-5.4 | DS-Fast | 1.250E-01 | 1.000E+00 |
| Basic Knowledge | General Introduction | 27 | GPT-5.4 | DS-Expert | 3.750E-01 | 1.000E+00 |
| Basic Knowledge | General Introduction | 27 | DS-Fast | DS-Expert | 1.000E+00 | 1.000E+00 |
| Basic Knowledge | Fundamentals of Radiotherapy Physics | 137 | GPT-4o | GPT-5.4 | 4.966E-01 | 1.000E+00 |
| Basic Knowledge | Fundamentals of Radiotherapy Physics | 137 | GPT-4o | DS-Fast | 3.388E-21 | 2.982E-19 |
| Basic Knowledge | Fundamentals of Radiotherapy Physics | 137 | GPT-4o | DS-Expert | 1.694E-21 | 1.525E-19 |
| Basic Knowledge | Fundamentals of Radiotherapy Physics | 137 | GPT-5.4 | DS-Fast | 3.578E-18 | 2.970E-16 |
| Basic Knowledge | Fundamentals of Radiotherapy Physics | 137 | GPT-5.4 | DS-Expert | 1.084E-19 | 9.324E-18 |
| Basic Knowledge | Fundamentals of Radiotherapy Physics | 137 | DS-Fast | DS-Expert | 1.000E+00 | 1.000E+00 |
| Basic Knowledge | Fundamentals of Radiotherapy Biology | 92 | GPT-4o | GPT-5.4 | 4.406E-08 | 2.952E-06 |
| Basic Knowledge | Fundamentals of Radiotherapy Biology | 92 | GPT-4o | DS-Fast | 2.354E-14 | 1.812E-12 |
| Basic Knowledge | Fundamentals of Radiotherapy Biology | 92 | GPT-4o | DS-Expert | 2.220E-16 | 1.732E-14 |
| Basic Knowledge | Fundamentals of Radiotherapy Biology | 92 | GPT-5.4 | DS-Fast | 1.312E-03 | 6.299E-02 |
| Basic Knowledge | Fundamentals of Radiotherapy Biology | 92 | GPT-5.4 | DS-Expert | 1.526E-05 | 9.155E-04 |
| Basic Knowledge | Fundamentals of Radiotherapy Biology | 92 | DS-Fast | DS-Expert | 2.500E-01 | 1.000E+00 |
| Relevant Professional Knowledge | Head and Neck Tumors | 60 | GPT-4o | GPT-5.4 | 1.000E+00 | 1.000E+00 |
| Relevant Professional Knowledge | Head and Neck Tumors | 60 | GPT-4o | DS-Fast | 5.188E-04 | 2.542E-02 |
| Relevant Professional Knowledge | Head and Neck Tumors | 60 | GPT-4o | DS-Expert | 6.104E-05 | 3.418E-03 |
| Relevant Professional Knowledge | Head and Neck Tumors | 60 | GPT-5.4 | DS-Fast | 1.221E-04 | 6.470E-03 |
| Relevant Professional Knowledge | Head and Neck Tumors | 60 | GPT-5.4 | DS-Expert | 6.104E-05 | 3.418E-03 |
| Relevant Professional Knowledge | Head and Neck Tumors | 60 | DS-Fast | DS-Expert | 1.000E+00 | 1.000E+00 |
| Relevant Professional Knowledge | Thoracic Tumors | 56 | GPT-4o | GPT-5.4 | 1.000E+00 | 1.000E+00 |
| Relevant Professional Knowledge | Thoracic Tumors | 56 | GPT-4o | DS-Fast | 1.172E-02 | 5.156E-01 |
| Relevant Professional Knowledge | Thoracic Tumors | 56 | GPT-4o | DS-Expert | 1.953E-03 | 9.180E-02 |
| Relevant Professional Knowledge | Thoracic Tumors | 56 | GPT-5.4 | DS-Fast | 1.172E-02 | 5.156E-01 |
| Relevant Professional Knowledge | Thoracic Tumors | 56 | GPT-5.4 | DS-Expert | 1.953E-03 | 9.180E-02 |
| Relevant Professional Knowledge | Thoracic Tumors | 56 | DS-Fast | DS-Expert | 1.000E+00 | 1.000E+00 |
| Relevant Professional Knowledge | Abdominal Tumors | 51 | GPT-4o | GPT-5.4 | 3.018E-01 | 1.000E+00 |
| Relevant Professional Knowledge | Abdominal Tumors | 51 | GPT-4o | DS-Fast | 2.891E-01 | 1.000E+00 |
| Relevant Professional Knowledge | Abdominal Tumors | 51 | GPT-4o | DS-Expert | 1.563E-02 | 6.563E-01 |
| Relevant Professional Knowledge | Abdominal Tumors | 51 | GPT-5.4 | DS-Fast | 3.516E-02 | 1.000E+00 |
| Relevant Professional Knowledge | Abdominal Tumors | 51 | GPT-5.4 | DS-Expert | 4.883E-04 | 2.490E-02 |
| Relevant Professional Knowledge | Abdominal Tumors | 51 | DS-Fast | DS-Expert | 2.500E-01 | 1.000E+00 |
| Relevant Professional Knowledge | Cervical Cancer | 8 | GPT-4o | GPT-5.4 | 1.000E+00 | 1.000E+00 |
| Relevant Professional Knowledge | Cervical Cancer | 8 | GPT-4o | DS-Fast | 1.000E+00 | 1.000E+00 |
| Relevant Professional Knowledge | Cervical Cancer | 8 | GPT-4o | DS-Expert | 1.000E+00 | 1.000E+00 |
| Relevant Professional Knowledge | Cervical Cancer | 8 | GPT-5.4 | DS-Fast | 1.000E+00 | 1.000E+00 |
| Relevant Professional Knowledge | Cervical Cancer | 8 | GPT-5.4 | DS-Expert | 1.000E+00 | 1.000E+00 |
| Relevant Professional Knowledge | Cervical Cancer | 8 | DS-Fast | DS-Expert | 1.000E+00 | 1.000E+00 |
| Professional Knowledge | Machines and Auxiliary Equipment | 118 | GPT-4o | GPT-5.4 | 1.000E+00 | 1.000E+00 |
| Professional Knowledge | Machines and Auxiliary Equipment | 118 | GPT-4o | DS-Fast | 1.455E-11 | 1.077E-09 |
| Professional Knowledge | Machines and Auxiliary Equipment | 118 | GPT-4o | DS-Expert | 2.838E-10 | 2.043E-08 |
| Professional Knowledge | Machines and Auxiliary Equipment | 118 | GPT-5.4 | DS-Fast | 1.455E-11 | 1.077E-09 |
| Professional Knowledge | Machines and Auxiliary Equipment | 118 | GPT-5.4 | DS-Expert | 2.838E-10 | 2.043E-08 |
| Professional Knowledge | Machines and Auxiliary Equipment | 118 | DS-Fast | DS-Expert | 1.000E+00 | 1.000E+00 |
| Professional Knowledge | Radiotherapy Process | 52 | GPT-4o | GPT-5.4 | 2.891E-01 | 1.000E+00 |
| Professional Knowledge | Radiotherapy Process | 52 | GPT-4o | DS-Fast | 9.537E-07 | 6.008E-05 |
| Professional Knowledge | Radiotherapy Process | 52 | GPT-4o | DS-Expert | 9.537E-07 | 6.008E-05 |
| Professional Knowledge | Radiotherapy Process | 52 | GPT-5.4 | DS-Fast | 1.526E-05 | 9.155E-04 |
| Professional Knowledge | Radiotherapy Process | 52 | GPT-5.4 | DS-Expert | 1.526E-05 | 9.155E-04 |
| Professional Knowledge | Radiotherapy Process | 52 | DS-Fast | DS-Expert | 1.000E+00 | 1.000E+00 |
| Professional Knowledge | Irradiation Techniques and Field Design | 48 | GPT-4o | GPT-5.4 | 4.545E-01 | 1.000E+00 |
| Professional Knowledge | Irradiation Techniques and Field Design | 48 | GPT-4o | DS-Fast | 4.883E-04 | 2.490E-02 |
| Professional Knowledge | Irradiation Techniques and Field Design | 48 | GPT-4o | DS-Expert | 1.907E-06 | 1.163E-04 |
| Professional Knowledge | Irradiation Techniques and Field Design | 48 | GPT-5.4 | DS-Fast | 4.425E-03 | 1.991E-01 |
| Professional Knowledge | Irradiation Techniques and Field Design | 48 | GPT-5.4 | DS-Expert | 3.052E-05 | 1.740E-03 |
| Professional Knowledge | Irradiation Techniques and Field Design | 48 | DS-Fast | DS-Expert | 2.500E-01 | 1.000E+00 |
| Professional Knowledge | IMRT and SRT | 100 | GPT-4o | GPT-5.4 | 1.000E+00 | 1.000E+00 |
| Professional Knowledge | IMRT and SRT | 100 | GPT-4o | DS-Fast | 4.172E-07 | 2.670E-05 |
| Professional Knowledge | IMRT and SRT | 100 | GPT-4o | DS-Expert | 2.980E-08 | 2.027E-06 |
| Professional Knowledge | IMRT and SRT | 100 | GPT-5.4 | DS-Fast | 1.192E-07 | 7.749E-06 |
| Professional Knowledge | IMRT and SRT | 100 | GPT-5.4 | DS-Expert | 5.960E-08 | 3.934E-06 |
| Professional Knowledge | IMRT and SRT | 100 | DS-Fast | DS-Expert | 1.000E+00 | 1.000E+00 |
| Professional Knowledge | Quality Assurance | 90 | GPT-4o | GPT-5.4 | 8.642E-01 | 1.000E+00 |
| Professional Knowledge | Quality Assurance | 90 | GPT-4o | DS-Fast | 5.378E-17 | 4.302E-15 |
| Professional Knowledge | Quality Assurance | 90 | GPT-4o | DS-Expert | 8.674E-19 | 7.373E-17 |
| Professional Knowledge | Quality Assurance | 90 | GPT-5.4 | DS-Fast | 2.082E-16 | 1.645E-14 |
| Professional Knowledge | Quality Assurance | 90 | GPT-5.4 | DS-Expert | 3.469E-18 | 2.914E-16 |
| Professional Knowledge | Quality Assurance | 90 | DS-Fast | DS-Expert | 5.000E-01 | 1.000E+00 |
| Professional Practical Ability | Responsibilities of Radiation Technician | 37 | GPT-4o | GPT-5.4 | 1.000E+00 | 1.000E+00 |
| Professional Practical Ability | Responsibilities of Radiation Technician | 37 | GPT-4o | DS-Fast | 6.250E-02 | 1.000E+00 |
| Professional Practical Ability | Responsibilities of Radiation Technician | 37 | GPT-4o | DS-Expert | 6.250E-02 | 1.000E+00 |
| Professional Practical Ability | Responsibilities of Radiation Technician | 37 | GPT-5.4 | DS-Fast | 3.125E-02 | 1.000E+00 |
| Professional Practical Ability | Responsibilities of Radiation Technician | 37 | GPT-5.4 | DS-Expert | 3.125E-02 | 1.000E+00 |
| Professional Practical Ability | Responsibilities of Radiation Technician | 37 | DS-Fast | DS-Expert | 1.000E+00 | 1.000E+00 |
| Professional Practical Ability | Simulation Localization Techniques | 76 | GPT-4o | GPT-5.4 | 2.471E-04 | 1.285E-02 |
| Professional Practical Ability | Simulation Localization Techniques | 76 | GPT-4o | DS-Fast | 2.776E-17 | 2.276E-15 |
| Professional Practical Ability | Simulation Localization Techniques | 76 | GPT-4o | DS-Expert | 2.776E-17 | 2.276E-15 |
| Professional Practical Ability | Simulation Localization Techniques | 76 | GPT-5.4 | DS-Fast | 9.313E-10 | 6.519E-08 |
| Professional Practical Ability | Simulation Localization Techniques | 76 | GPT-5.4 | DS-Expert | 9.313E-10 | 6.519E-08 |
| Professional Practical Ability | Simulation Localization Techniques | 76 | DS-Fast | DS-Expert | 1.000E+00 | 1.000E+00 |
| Professional Practical Ability | Positioning Techniques | 101 | GPT-4o | GPT-5.4 | 6.575E-05 | 3.550E-03 |
| Professional Practical Ability | Positioning Techniques | 101 | GPT-4o | DS-Fast | 6.776E-21 | 5.895E-19 |
| Professional Practical Ability | Positioning Techniques | 101 | GPT-4o | DS-Expert | 1.694E-21 | 1.525E-19 |
| Professional Practical Ability | Positioning Techniques | 101 | GPT-5.4 | DS-Fast | 1.000E-11 | 7.503E-10 |
| Professional Practical Ability | Positioning Techniques | 101 | GPT-5.4 | DS-Expert | 2.274E-13 | 1.728E-11 |
| Professional Practical Ability | Positioning Techniques | 101 | DS-Fast | DS-Expert | 5.000E-01 | 1.000E+00 |
